# Loss of the aryl hydrocarbon receptor promotes cancer cell resistance to BRAFV600E targeted therapies

**DOI:** 10.1038/s41392-025-02235-6

**Published:** 2025-05-14

**Authors:** Mourad Zerfaoui, Dharmeshkumar Patel, Yueqi Zhang, Raymond F. Schinazi, Youssef Errami

**Affiliations:** 1 Center for ViroScience and Cure, Laboratory of Biochemical Pharmacology, Department of Pediatrics, Emory University School of Medicine and Children’s Healthcare of Atlanta, 1760 Haygood Dr, HSRB1, Atlanta, GA 30322 USA; 2 Department of Neurosurgery, Xiangya Hospital, Central South University, Changsha, 410008 China; 3 Department of Neuroscience, Yale School of Medicine, Yale University, New Haven, CT 06511 USA; 4 Department of Microbiology, Immunology, and Molecular Genetics, University of California, Los Angeles, CA 90095 USA

**Keywords:** Cancer therapy, Translational research

**Dear Editor**,

Predicting a patient’s response to cancer therapies and identifying additional molecular targets is crucial for advancing cancer treatment. BRAF^V600E^-specific inhibitors have demonstrated promising initial clinical results in solid tumors; however, side effects and drug resistance are common, and for most patients, response to BRAF^V600E^ inhibitor therapy is transient. Treatment with dabrafenib (Dab), a selective BRAF^V600E^ inhibitor, in combination with trametinib (Tra), a MEK1/2 inhibitor, is effective for BRAF^V600E+^ unresectable or metastatic solid tumors. However, resistance mechanisms and activation of alternative proliferation pathways can still bypass BRAF/MEK dual blockade.^1^

To comprehensively identify genes that regulate resistance to MAPK inhibitors, we selected two well-characterized human thyroid cancer (TC) cell lines, validated for preclinical studies, namely K1 (GLAG-66) and 8505c. Both cell lines are BRAF^V600E^-positive, but each has its own distinct clinical characteristics and genomic alterations.^2^ K1 and 8505c cell lines stably expressing spCas9 were transduced with a second lentiviral vector containing the sgRNA cassette (Fig. 1a). To study combination Dab+Tra (D + T) therapy in these cell lines, we first determined their inhibitory concentration dosage curves and selected a combination dosage of 10 μM Dab + 10 nM Tra for the screen. The Brunello CRISPR knockout lentiviral genome-wide library was used to identify genes whose loss confers resistance or sensitization to MAPK inhibitors. We analyzed and determined the statistical significance of each sgRNA change. Among the top scorers, with a FDR below 5% for both cell lines, we found genes known to cause resistance against MAPK inhibitors (*NF1*, *CUL3*, *NF2*, and *MED12*),^3^ validating our screening and methodology. We also found that in both 8505c and K1, *AHR* was highly ranked (Fig. 1a). AhR has been studied extensively due to its central role in autoimmune disorders, inflammatory diseases, endocrine disruption, premature aging, and cancer, among other conditions, but its role in MAPK inhibitor resistance in TC is not well understood.

**Fig. 1:**
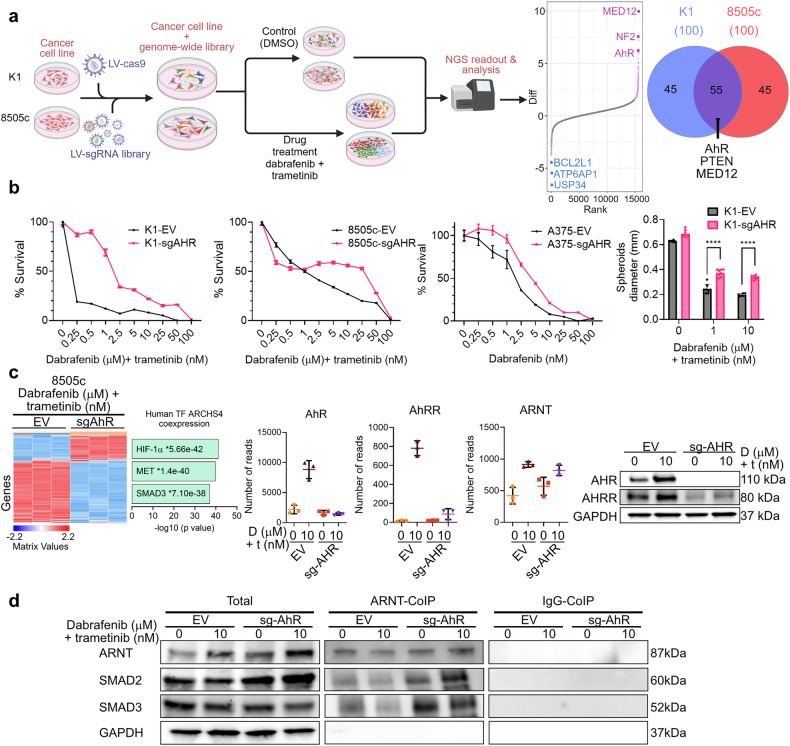
**a** Experimental workflow for the in vitro genetic knock-out screen in K1 and 8505c cell lines. K1 and 8505c cells were transduced with lentiviruses from the Brunello plasmid library and selected with puromycin for 7 days. Afterward, cells were treated with 10 μM dabrafenib and 10 nM trametinib. Samples were collected on day 21 or at full plate confluency. gDNA was isolated, and sgRNA abundance was assessed by PCR amplification of sgRNA sequences from gDNA, followed by NGS (diagram generated using BioRender). The MAGeCK plot displays normalized CRISPR viability scores for genes targeted by sgRNAs. The *x*-axis represents sensitivity or resistance, calculated as the mean of all four sgRNAs for each gene in the sorted population versus the unsorted population. The *y*-axis indicates statistical significance via the FDR-corrected *p* value. A Venn diagram represents the top 100 hits of K1 and 8505, intersecting genes. **b** A luciferase assay evaluates survival levels after 4 days of treatment. The 8505c, K1, and A375 cell lines are seeded at 2000 cells/well in 96-well plates and treated with varying concentrations of trametinib and dabrafenib for 4 days, with cell viability calculated as a percentage of the cells treated with 0.1% DMSO. The sizes of K1 spheroids are quantified by measuring diameters. Data are shown as the mean ± s.e.m. Error bars represent SD. (*n* = 6, ****p 0.001). **c** Differentially expressed genes in 8505c-EV cells were compared to 8505c-AhR-KO cells after 4 days of treatment with 10 μM Dabrafenib and 10 nM Trametinib. A heatmap was generated using ClusterGrammar after normalizing the raw gene counts via the logCPM method, filtering for the 2500 most variable genes, and transforming the data using the *Z*-score method. mRNA reads from RNA-seq were collected for AhR, AhRR, and ARNT. Western blots of total cell lysate from 8505c confirmed the RNA-seq data. Transcription Factor Enrichment Analysis was conducted on upregulated and downregulated gene sets using Enrichr with the ARCHS4_TFs_Coexp library. Significant results were established with a *p* value cut-off of 0.1 after Benjamini–Hochberg correction. **d** Whole-cell lysates from 8505c cells (control Empty Vector and 8505c AhR knock-out) were treated or untreated with the drug combination and immunoprecipitated with an ARNT-specific antibody

To confirm and validate the effects of AhR-targeted therapies on TC and melanoma cell survival, we used K1, 8505c, and A375 cells stably expressing spCas9 and a sgRNA targeting AhR (sgAhR). The same plasmid construct without sgRNA was used as a control empty vector (EV). A luciferase-based cell proliferation assay validated that AhR knockout cells are less sensitive to treatment with MAPK inhibitors after four days compared to the treated NTC control cells, confirming that AhR loss-of-function offers an escape from targeted therapies in both TC cell lines. Using K1 spheroids, we also found that AhR knockout spheroids were significantly more resistant to drug treatment compared to the treated NTC control spheroids. Together, these data confirm that genetic suppression of AhR induces resistance to MAPK inhibitors in BRAF^V600E^-positive cancers (Fig. 1b).

To understand how the loss of AhR influences treatment resistance in 8505c cancer cells, we established the transcriptome profile of 8505c NTC and AhR-KO cells treated for four days with a D + T combination. AhR repressor (AhRR) is a feedback regulator of AhR activity. RNA-seq analysis reveals that D + T treatment elevates the expression of both AhR and AhRR (Fig. 1c). As expected, western blot confirmed that CRISPR-Cas9 knockout of AhR diminishes AhRR protein levels. ARNT gene expression is not affected by the loss of AhR. Gene Set Enrichment Analysis (GSEA) of transcription factor signature genes reveals the expression of SMAD3-dependent genes, suggesting that the loss of AhR may directly or indirectly activate the TGF-β/SMAD proliferation pathway and provide an escape route for targeted therapies (Fig. 1c).

Upon binding to its ligand, AhR translocates into the nucleus, dimerizes with ARNT, and regulates the transcription of genes containing the AhR-responsive elements in their promoters. AhR regulates genes that control drug metabolism, AhRR, and cell proliferation, differentiation, and apoptosis. AhRR also dimerizes with ARNT, interfering competitively with AhR-ARNT complex formation and repressing AhR-regulated gene expression. The loss of AhR does not affect ARNT protein expression. ARNT can also form heterodimers with various proteins, including HIF-1α and SMADs.^4^ Our GSEA identified unexpectedly elevated HIF-1α-dependent gene expression in AhR knockout cells treated with targeted therapies. No significant alterations in phosphorylation levels were detected in proliferation-inducing pathways after the loss of AhR. We hypothesized that ARNT could also form a dimer with SMAD2 and SMAD3, acting as a co-transcription factor. Indeed, ARNT co-immunoprecipitation pulled down higher levels of SMAD3 and SMAD2 proteins in AhR knockout cells (Fig. 1d). Molecular docking analysis^5^ of AhR, SMAD2, SMAD3, and HIF1-α with ARNT, as well as binding affinity calculations of each complex, show that AhRR has binding affinity for ARNT of ΔG = −516.18 kcal/mol, AhR has ΔG = −314.48 kcal/mol, SMAD3 has ΔG = −168.95 kcal/mol, and SMAD2 ΔG = −132.03 kcal/mol. In contrast, HIF-1α has ΔG =−124.66 kcal/mol. An analysis of structural binding affinity confirmed the affinity of SMAD2 and SMAD3 for the ARNT co-transcription factor.

In summary, the loss of AhR provides cancer cells with an escape from MAPK blockade through the TGF-β/SMAD pathway. Investigation of possible mechanisms revealed that SMAD2/3 competes with AhR for binding to ARNT. When AhR is absent, the interaction between ARNT and SMAD2/3 increases, leading to higher expression of TGF-β-dependent genes. This suggests a remarkably intricate regulatory complex, with ARNT at its center, in which AhR, AhRR, HIF-1α, SMAD2/3, and others compete to form heterodimers. Molecular docking suggests that the binding priority for ARNT is AhRR > AhR > SMAD3 > SMAD2 > HIF-1α. Further studies into the binding partners and transcriptional activity of ARNT may reveal a mechanism of gene expression that balances the interplay between transcription factors, influencing drug resistance and cell function.

## Supplementary information

Supplementary Materials

## Data Availability

Raw data are publicly available through GEO Series accession numbers GSE286107 and GSE286108. Analyses were performed using openly available software and toolboxes as described in supplementary materials.
