# Supplementary material for: Loss of the aryl hydrocarbon receptor promotes cancer cell resistance to BRAFV600E targeted therapies
Source: Signal Transduct Target Ther. 2025 May 14;10:158. doi: 10.1038/s41392-025-02235-6 (PMC12075852; doi:10.1038/s41392-025-02235-6)
Supplement: Supplementary file 1 — Supplementary Materials [file 41392_2025_2235_MOESM1_ESM.docx]

**Supplementary Materials for:**

**Loss of the Aryl Hydrocarbon Receptor promotes cancer cell resistance to BRAF^V600E^ targeted therapies.**

Mourad Zerfaoui*^1^, Patel Dharmeshkumar Jethalal^1^, Yueqi Zhang^2,3^, Raymond F. Schinazi^1^, Youssef Errami*^4^

**Material and Methods.**

**Cell Lines and Reagent.**

Three human cancer cell lines, K1, 8505c, and A375, were used in this study. K1 and 8505c were purchased from Millipore-Sigma Cat. No. 92030501, and Cat. No. 94090184. A375 was from the American Type Culture Collection (ATCC) VA, USA. Cells were cultured according to provider instructions. Cell lines used in this study were below 20 passages. For packaging viruses, HEK293T cells were purchased from ATCC (Cat. No. CRL-3216). All cell lines were mycoplasma-free. Dabrafenib (Cat. No. S2807) and Trametinib (Cat. No. S2673) were purchased from Selleck Chemicals, TX, USA. Stock solutions were prepared in DMSO and stored at -20°C.

**Library and Construct.**

Human Brunello CRISPR knockout pooled library was a gift from David Root and John Doench (Addgene #73178). lentiCas9-Blast was a gift from Feng Zhang (Addgene plasmid # 52962). pLenti-U6sgbbBsmbI-puro-2A-Fluc was a gift from Sidi Chen (Addgene plasmid # 100277). The standard protocols to anneal and clone the sgRNA oligos into the lentiCRISPR v2 vector.

**Virus production and infection.**

To produce Lentiviruses, HEK293T cells were plated in a 15-cm tissue culture plate. Once confluency is reached media was replaced with 15ml OPTI-MEM 1-2 hours before transfection. The transfection mix was typically composed of 20 μg vector DNA, 15 μg psPAX2 packaging plasmid (Addgene # 12260), 10 μg pMD2.G (Addgene # 12259) envelope plasmid, 440 μl of OPTI MEM, and 130 μl transfection reagent PEI MAX Polysciences PA, USA (Cat. No. 24765-100). The multiplicity of infection (MOI) was determined for each batch of produced lentiviruses and for each individual cell line by infecting cells with incremental amounts of lentivirus by Puromycin (10 μg/ml) selection over two days. Numbers of surviving cells were counted and compared to the control wells, a survival rate of 30% was considered to be a MOI of 0.3.

**Luciferase-based survival assay**

Cells transduced with the lentivirus selected by puromycin and expressing the luciferase transgene were seeded into 100 μl of medium at a density of 1,000–2,000 cells per well in white flat-bottom 96 well plates Greiner Bio NC, USA (Cat. No. 655983). Treatment using inhibitors started 24 hours after seeding. 4 days after the beginning of the treatment, cell viability was assessed using D-luciferin Revvity MA, USA (Cat. No. 122799), and luminescence was quantified using the BioTek Cytation 5 plate reader. The cell viability of wells containing DMSO‐treated cells was set as 100% all other measures were normalized to these values.

**Pooled CRISPR screen**

For the pooled CRISPR screen, a total of 1×10^8^ cells were infected with the pooled lentiviral library at a multiplicity of infection (MOI) of 0.3. After puromycin selection, the cells were divided into two groups (Day 0 and treatment). The cell pellet of Day 0 group was stored at −80 °C. For the treatment groups, the cells were cultured for 21 days or until confluency was reached and treated with 10 μM of Dabrafenib+ 10nM of Trametinib. Media was replaced with fresh media every 3-4 days.

**Amplification and sequencing of sgRNAs from cells.**

After harvesting the cells from different groups, we extracted the genomic DNA. Library construction for NGS was performed by two-step PCR. The PCR products were purified and then sequenced on a HiSeq 2500 by Azenta Next Generation Sequencing Services. Each library was sequenced at 30–40 million reads to achieve ∼300 × average coverage over the CRISPR library.

**CRISPR screen analysis.**

The CRISPR/Cas9 screening data were performed by MAGeCK and MAGeCK-VISPR algorithms. MAGeCK-VISPR calculated the beta score for each gene. The differential beta scores between the BRAFi treatment and DMSO treatment were compared using MAGeCKFlute, which was designed to perform quality control, normalization, and downstream analysis of the functional CRISPR screens.

**Single sgRNA knockout lentiviral production.**

For the cloning of sgRNA targeting AhR Fwd: CGTAAGATGGTTAGCTTGTC, the corresponding oligos were synthesized, annealed and cloned into BsmBI linearized pLenti-U6sgbbBsmbI-puro-2A-Fluc lentiviral knockout vector.

**Isolation of total RNA.**

The cell pellets were collected by scraping, and the total RNA was isolated using the QIAGEN RNeasy Purification Kit (Cat. No. 74104), following the manufacturer’s instructions. TruSeq library preparation and paired-end sequencing (2 × 100 bp) on the Illumina NextSeq 2000 instrument (Illumina) were performed at the Genomics Core at the Tulane Center for Aging. Around 100 million total reads were obtained for each sample.

**RNASeq data analysis.**

The Ensembl GRCh38.p14 reference genome was used to map genes, and the RASflow RNA sequencing workflow was used for analysis. Normalized Raw counts were used to generate the gene expression signature by comparing gene expression levels between the control 8505c cells and the AhR-generated knock-out cells group. The heatmap was generated using clustergrammer. Before displaying the heatmap, the raw gene counts were normalized using the logCPM method, filtered by selecting the 2500 genes with the most variable expression, and finally transformed using the Z-score method. The Augmented Gene Set Enrichment Analysis (AGSEA) Appyter was used to perform and visualize standard gene set enrichment analysis (GSEA). Wikipathways was the chosen gene set library selected. The Transcription Factor Enrichment Analysis Enrichment results were generated by analyzing the up-regulated and down-regulated gene sets using Enrichr. The following libraries were used for the analysis: ARCHS4_TFs_Coexp. Significant results are determined by using a cut-off of p-value<0.1 after applying the Benjamini-Hochberg correction. The same filtered gene of the 2500 most differentiated was used for the transcription factor target over-representation analysis using ChEA3.

**Spheroid formation assay**

Spheroids were produced by seeding in each well 10,000 control EV or AhR knockout K1 cells in ultra-low attachment 96 well plates (Corning 4515) with media supplemented by 1% rat tail collagen (Gibco A1048301).

**Immunoblotting.**

Whole-cell extracts for Western-Blot were prepared by incubating cells on ice in RIPA lysis buffer plus Halt Protease and phosphatase inhibitors Thermo Fisher Scientific MA, USA (Cat. No. 78440) for 30 min. After centrifugation (>15,000 r.c.f., 10 min, 4 °C), protein lysates were quantitated using the Pierce BCA Protein Assay Kit Thermo (Cat. No. 23225). Lysates were fractionated in 4–20% precast polyacrylamide gel Bio-Rad CA, USA (Cat. No. 4561094) and transferred to a PVDF membrane Millipore Sigma (Cat. No. IEVH07850). The membranes were blocked for 1 h in 10% milk TBST buffer. List of Antibodies used in Supplementary Table 3. The secondary antibodies Peroxidase AffiniPure Goat Anti-Mouse IgG (H+L) and Anti-Rabbit IgG (H+L) Jackson ImmunoResearch PA, USA (Cat. No. 115-035-003 and 111-035-003) were used for immunofluorescence staining. Membrane imaging was performed using Bio-Rad Chemidoc.

**Co-immunoprecipitation assays.**

Cell lysates were prepared using the ice-cold Pierce IP Lysis Buffer (Thermo Fisher Scientific) for 15 min at 4°C, followed by centrifugation at 10,000 g for 10 min. The supernatant (Input) was transferred to a new tube and immediately aliquoted for the BCA assay to determine protein concentration. All steps were conducted at 4°C to stabilize and detect complex formation. Co-IP was performed using the Thermo Scientific Pierce Crosslink Magnetic IP/Co-IP Kit (Thermo Fisher Scientific Cat. No. 88805) according to the manufacturer’s instructions. First, the beads were pre-washed with 1X Coupling Buffer. Then, 10 µg ARNT antibody or control IgG antibody were diluted to a final volume of 100 µL and bound to Protein A/G magnetic beads for 15 min. The beads were washed three times with 1X Coupling Buffer, and the antibody was cross-linked to the beads with disuccinimidyl suberate (DSS) for 30 min. The beads were then washed three times with Elution Buffer and twice with IP Lysis/Wash Buffer. The lysate solution was diluted with IP Lysis/Wash Buffer to 200 µL with a total protein concentration of 300 μg and incubated with prepared beads overnight at 4°C. The beads were washed twice with IP Lysis/Wash Buffer and then once with purified water. The bound antigen was eluted with 100 µL of Elution Buffer and incubated for 5 min at room temperature on a rotator. The beads were magnetically separated, and the supernatant containing the target antigen was saved. The supernatant was diluted with 4x Laemmli Sample buffer (Bio-Rad), and 15 μL of sample was loaded onto SDS-PAGE for western blotting.

**Statistical analysis.**

Data are presented as mean ± standard deviation (SD) or standard error of the mean (SEM) from at least three independent experiments. Statistical significance was determined using a two-tailed Student's t-test or one-way ANOVA followed by Tukey's post hoc test, as appropriate. P-values < 0.05 were considered statistically significant. All statistical analyses were performed using GraphPad Prism 10 (GraphPad Software).

**Molecular docking and binding affinity predictions**

The docking analysis followed a similar methodology described previously. Briefly, the three-dimensional structures of AhRR with ARNT (PDB: 5Y7Y), AhR (PDB: 5V0L), HIF-1A (PDB: 7A1Q), SAMD2 MH2 domain(PDB: 6ZVQ) and SMAD3 MH2 domain (PDB: 1MK2) proteins were downloaded from the Protein Data Bank (PDB; <http://www.rcsb.org/>) and prepared using Protein Preparation Wizard in the Schrödinger suite. The protein-protein docking was performed using the PIPER module (Schrödinger Release 2024–2: PIPER, Schrödinger, LLC, New York, NY, 2024) present in the Schrödinger suite. PIPER algorithm searched for the 30 best complexes from 70,0000 possible protein-protein configurations. The standard mode selected with refine output poses and reserve archive of top 1000 clustered poses. The topmost pose of each complex was selected for binding affinity calculations. The binding affinity was predicted using the Prime MM-GBSA module of the Schrödinger suite. VSGB (variable-dielectric generalized Born) solvation model and OPLS3e force field were utilized during Prime MM-GBSA calculations. For the sampling, the “minimize” option was selected.

**Antibody List.**

SMAD2 (D43B4) XP® Rabbit mAb: 5339T; Cell Signaling Technology. SMAD3 (C67H9) Rabbit mAb: 9523T; Cell Signaling Technology. Normal Rabbit IgG: 2729S; Cell Signaling Technology.

HIF-1β/ARNT (D28F3) XP® Rabbit mAb: 5537S; Cell Signaling Technology. GAPDH Antibody (6C5): sc-32233; Santa-Cruz Biotechnology.

**Data availability.**

The data are publicly available through GEO Series accession numbers GSE286107 and GSE286108.
